# The Development of Technology to Prevent, Diagnose, and Manage Antimicrobial Resistance in Healthcare-Associated Infections

**DOI:** 10.3390/vaccines10122100

**Published:** 2022-12-08

**Authors:** Ayman Elbehiry, Eman Marzouk, Adil Abalkhail, Yasmine El-Garawany, Sulaiman Anagreyyah, Yaser Alnafea, Abdulaziz M. Almuzaini, Waleed Alwarhi, Mohammed Rawway, Abdelmaged Draz

**Affiliations:** 1 Department of Public Health, College of Public Health and Health Informatics, Qassim University, Al Bukayriyah 52741, Saudi Arabia; 2 Department of Bacteriology, Mycology and Immunology, Faculty of Veterinary Medicine, University of Sadat City, Sadat City 32511, Egypt; 3 Clinical Pharmacy Program, Faculty of Pharmacy, Alexandria University, Alexandria 21521, Egypt; 4 Department of Preventive Medicine, King Fahad Armed Hospital, Jeddah 23311, Saudi Arabia; 5 Department of Statistics, King Fahad Armed Hospital, Jeddah 23311, Saudi Arabia; 6 Department of Veterinary Medicine, College of Agriculture and Veterinary Medicine, Qassim University, Buraydah 52571, Saudi Arabia; 7 Department of Botany and Microbiology, College of Science, King Saud University, Riyadh 11451, Saudi Arabia; 8 Biology Department, College of Science, Jouf University, Sakaka 42421, Saudi Arabia; 9 Botany and Microbiology Department, Faculty of Science, Al-Azhar University, Assiut 71524, Egypt

**Keywords:** antimicrobial resistance, multidrug-resistant bacteria, rapid detection, alternative therapy, prevention

## Abstract

There is a growing risk of antimicrobial resistance (AMR) having an adverse effect on the healthcare system, which results in higher healthcare costs, failed treatments and a higher death rate. A quick diagnostic test that can spot infections resistant to antibiotics is essential for antimicrobial stewardship so physicians and other healthcare professionals can begin treatment as soon as possible. Since the development of antibiotics in the last two decades, traditional, standard antimicrobial treatments have failed to treat healthcare-associated infections (HAIs). These results have led to the development of a variety of cutting-edge alternative methods to combat multidrug-resistant pathogens in healthcare settings. Here, we provide an overview of AMR as well as the technologies being developed to prevent, diagnose, and control healthcare-associated infections (HAIs). As a result of better cleaning and hygiene practices, resistance to bacteria can be reduced, and new, quick, and accurate instruments for diagnosing HAIs must be developed. In addition, we need to explore new therapeutic approaches to combat diseases caused by resistant bacteria. In conclusion, current infection control technologies will be crucial to managing multidrug-resistant infections effectively. As a result of vaccination, antibiotic usage will decrease and new resistance mechanisms will not develop.

## 1. Introduction

Public health concerns about antimicrobial resistance (AMR) are growing worldwide, and it is increasingly recognized as a problem of global significance [1]. Infections caused by bacteria, viruses, or fungi can develop AMR in response to the introduction of antimicrobial therapy [2]. The importance of addressing this widespread and sophisticated hazard to human health has been highlighted in countless policy studies on an international and national scale [3]. As this review will solely cover bacterial resistance, readers are encouraged to examine other reviews regarding viral and fungal resistance [4,5,6]. AMR, which occurs when bacteria evolve in such a way that they become resistant to the medications used to treat diseases, poses one of the greatest risks to public health in the twenty-first century [7].

AMR is predicted to result in the deaths of ten million people a year by 2050, according to the United Kingdom Government’s Report on Bacterial Resistance [8,9], though other researchers have disputed these predictions [10]. It is essential to have a coordinated, worldwide action plan to prevent the spread of AMR, according to the WHO and other research organizations [11]. In order to understand bacterial AMR, the top pathogen–drug combinations, and current trends worldwide, it is critical to know the top pathogen–drug combinations. In the future, numerous pathogenic microorganisms might become much more deadly than they are today if AMR continues to develop uncontrolled [7]. The European Union (EU) considers AMR to be a worldwide issue and a top priority [12]. There are about 670,000 hospitalizations in the European Union/European Economic Area (EU/EEA) every year due to antimicrobial-resistant bacteria, and about 33,000 people die directly as a result [13], making AMR prevention and control initiatives crucial for Europeans.

Despite the uneven dispersion of the AMR matter across the globe [14], no nation can safely claim that it will not be impacted by the spreading of it. In his Nobel Prize acceptance speech, Fleming [15] advised that the world was heading for disaster after discovering penicillin, the first drug to be manufactured. In 1964, Fleming became aware of the dangers of AMR for everyone who wished to take these drugs, despite the fact that he did not anticipate the widespread of the infection.

It is challenging to identify the full cost of resistance when fighting AMR, especially in regions lacking good monitoring and statistics. Many investigations have been carried out in specific areas to predict the impacts of AMR on occurrence, mortalities, hospitalization lengths of stay, and insurance costs [16,17,18,19]. Despite this, no comprehensive forecasts have ever covered all places and a wide range of pathogenic organisms and drug combinations. According to Cassini and colleagues [17] of the European Union and the European Economic Area, eight bacterial pathogens and sixteen pathogen–drug combinations caused infections and deaths in 2007–2015, while the United States, the’ Centers for Disease Control and Prevention (CDC) published a 2019 article on AMR infections and deaths in the United States based on surveillance data [20]. Furthermore, in 2014, Temkin and colleagues estimated that *Escherichia coli* and *Klebsiella pneumoniae* were resistant to third-generation cephalosporins and carbapenems in 193 countries, while in 2010, Lim and colleagues reported that six pathogens in Thailand were resistant to multiple drugs [18].

Various mechanisms of action appear to be found in antimicrobial drugs, which are categorized by their chemical nature. Beta-lactams, fosfomycin, and vancomycin, for example, inhibit cell wall synthesis (beta-lactams); other antibiotics inhibit DNA replication (fluoroquinolones), protein synthesis (tetracyclines and aminoglycosides) and metabolic processes (trimethoprim and sulfonamides) [21]. Because of their cell wall construction, efflux systems, or porins, certain bacteria are inherently resistant to certain antibiotics [22]. Throughout this species, every strain is resistant to the specific drug. An emergence of resistance, however, occurs when some strains of the same species develop tolerance to an antimicrobial [2]. It has been shown that horizontal gene transfer can be used to acquire new antibiotic resistance genes or modulate existing genes, such as intracellular targets [23] or key metabolic genes [24]. Sun et al. [25] argue that horizontal gene transfer promotes the spread of antibiotic resistance genes intra- and inter-species and is one of the causes of AMR outbreaks [26]. Although antimicrobial drugs were explored and widely used long before antibiotic resistance genes were discovered [27,28], the widespread antibiotic resistance in low- and middle-income countries was caused mainly by improperly treating sewage, poor hygiene, and excessive use of antimicrobials in domesticated animal farms and healthcare settings [21,29,30].

Healthcare-associated infections (HAIs) are infections contracted in hospitals and other health care facilities. In most cases, they appear 48 h after hospitalization, however they can also appear after patients have been discharged [17,31]. In developing and advanced countries, these infections usually affect 7 to 10% of inpatients, respectively [32]. The number of people affected by HAI in Europe exceeds 3.2 million annually [33], with the patients’ immune status directly connected to the frequency and degree of infection. Hospitalized patients, neonates, organ recipients, and burn patients are the most impacted categories. Most patients admitted to the hospital have at least one HAI episode, according to a recent Serbian study. The WHO [34] reports that HAIs cause 70% of neonatal deaths in Southern Asia and Sub-Saharan Africa. Khan et al. [35] reported that the most frequent categories of HAI are breathing machine asthma (9–27%), circulatory infections related to catheters (12–25%), urinary infections related to catheters (12%), and wound infections (2–5%).

In 2017, the World Health Organization (WHO) divided the most dangerous bacteria associated with HAI into three categories based on their severity and global danger [36]. Worldwide, hospital-acquired infections are mostly caused by the ESKAPE pathogens, which can be classified as the first and the second categories of pathogenic bacteria. They include *Enterococcus faecium*, *Staphylococcus aureus*, *Klebsiella pneumoniae*, *Acinetobacter baumannii*, *Pseudomonas aeruginosa*, and *Enterobacter* species [37]. The ESKAPE infection has been linked to high mortality and morbidity rates in HAIs because it is resistant to a wide range of antibiotics, including last-resort antibiotics such as carbapenems and colistins. As a result, a new study from Greece found that carbapenem-resistant infections were associated with a doubled risk of mortality after 90 days [38]. Greece has the highest utilization of pharmaceuticals in the Eurozone, and the second-highest number of adjusted disability life years caused by AMR [39]. Multiple reports have described resistant bacteria in healthcare settings, particularly ESKAPE [40,41,42]. Researchers at a Singaporean hospital examined swabs from low- and high-contact areas, including bed posts, bedside storerooms, and door handles. In their paper, they document the alarming development and strength of multidrug resistant bacteria, some of which are combining brand-new and extremely risky antimicrobial resistance genes. In addition, the researchers discovered a methicillin-resistant plasmid (*mecA* gene) and an antiseptic tolerance plasmid. The fact that many strains isolated have remained in the hospital for almost ten years, causing secondary infections in hospitalized patients, is concerning [41].

Moreover, a wide variety of bacteria are capable of infecting a host and surviving there for a long period of time [43]. There are several factors responsible for this phenomenon, including a suppressed immune system, presence a pathogen that is able to evade the immune system, or antibiotic resistance [43]. As a result of their role in persistent infections, persister cells are now receiving attention. There are a number of pathogens that have persister cells well-documented within them, including *Salmonella enterica* serovar *Typhimurium*, *Escherichia coli*, *Pseudomonas aeruginosa*, *Mycobacterium tuberculosis*, and *Staphylococcus aureus* [44,45,46]. It is believed that they are responsible for the failure of antibiotic treatment [47,48] and might contribute to the emergence of antibiotic resistance [49], and therefore persisters are considered a serious public health issue as a result of their persistence [43].

**Figure 1:**
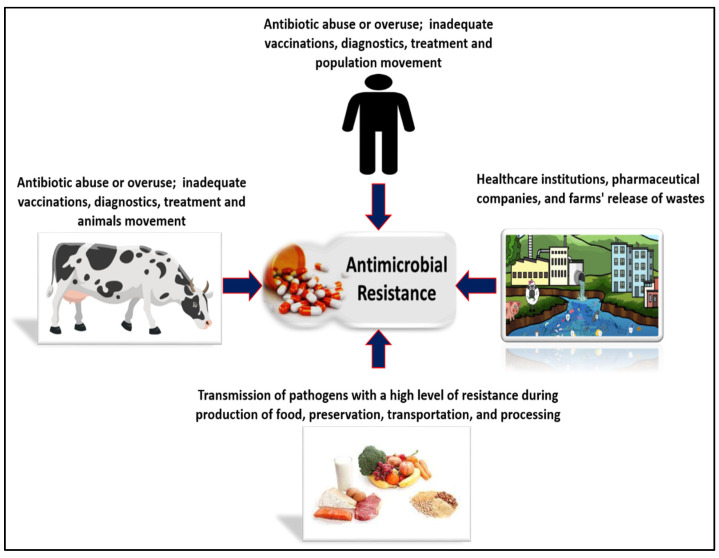
Components of the “One-Health Concept” to combat AMR.

Both people and animals can contract AMR from the surroundings. A bacterial population is released into the environment through human and animal feces. People can come in contact with these germs if they swim in water that is infected, consume tainted water, eat fresh fruit and vegetables, or breathe bioaerosols [50]. A previous study indicated that waterbodies offer a greater or similar risk of spreading *Salmonella* or *Campylobacter* than chicken eating. It has been estimated that the number of outbreaks of water-borne diseases caused by pathogenic *Escherichia coli* O157 in the United States is 9% [50]. In a recent report, the WHO estimated that raw or minimally fried seafood or exposure to coastal waters leads to over 120 million cases of digestive disorders each year [51]. As described in Figure 1, human, animal, plant, food, and environmental factors all contribute to the development of AMR, so a sustained One Health approach is necessary to bring all sectors of health and the environment together. A “One Health” approach involves designing and implementing programs, policies, legislation, and research that are intended to improve public health outcomes, as well as facilitating cooperation and communication between groups involved in food and feed production, the environment, animal and plant health on land and in the water, and human health.

**Figure 2:**
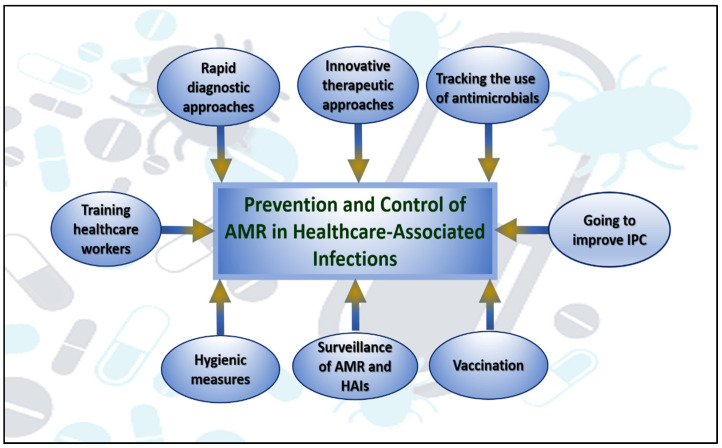
The fundamentals of AMR help improve the management of HAIs and reduce the negative impacts of antibiotic use.

Numerous studies have linked outbreaks of infection with Enterobacteriaceae to fresh food intake. In Europe and North America, *Escherichia coli* O104:H4 outbreaks linked to sprouts were reported in 2011, while in the United States, *Escherichia coli* O157:H7 outbreaks linked to spinach were reported in 2006. Several studies have shown that the environment can facilitate the spread of AMR bacteria to humans [52]. Environmental factors play a critical role in the spread of AMR bacteria to humans, as opposed to contact with animal carriers, eating animal products, traveling internationally, and spreading in healthcare and community settings, where these microorganisms can disperse [50]. To minimize the human influence on the emergence of AMR, a number of educational, regulatory, and political measures must be taken [53,54,55,56]. As shown in Figure 2, by implementing better cleaning and hygiene practices, we can reduce the prevalence of resistant bacteria. Moreover, we need to develop new, quick, and accurate instruments for diagnosing infections. Lastly, we need to explore new therapeutic approaches to combat diseases caused by resistant bacteria [2].

## 2. The Prevention of HAIs Caused by Multidrug-Resistant Microbes

As reported by Mata et al. [57], the preponderance of multidrug-resistant microorganisms is hospital in origin, and these bacteria are capable of persisting for decades inside healthcare settings [41]. Several studies have indicated that a patient who shares a room with a previous patient is more likely to contract HAI [58]. Healthcare facilities sanitation is seldom discussed when discussing AMR transmission, and current cleanliness practices have been largely unchanged during the past 25 years [59]. More than 180 nations now routinely use alcohol-based handrubs after the WHO adopted them in 2005 [60]. Nevertheless, two out of three institutions in less-developed countries lack acceptable waste disposal services, according to a recent WHO and UNICEF survey on health centers. At points of care, every third facility worldwide lacks appropriate hand hygiene [61]. The spread of antimicrobial-resistant bacteria, antimicrobial resistance genes, and genetic elements in the environment continues even after medical waste is treated [62]. In a recent study conducted by the German research team, they found that, despite similar median daily discharge rates, harmful byproducts from hospital wastewater treatment are likely to contain *NDM*-1, *mcr*-1, *vanA*, and *mecA* genes up to 70% higher than those from treating wastewater facilities for community or agricultural use [63]. Using hydrogen peroxide (H_2_O_2_), Wang et al. [64] describe electroperoxone (Eperoxone) as an effective method of treating waste water. By actively interacting with ozone, electrically generated H_2_O_2_ reduces the number of antimicrobial resistance genes [65]. The development of AMR is largely dependent on the increase in horizontal gene transfer in microorganisms induced by synthetic chemicals [66,67].

### 2.1. Managing Wastewater and Surfaces in Healthcare Settings

A number of chemicals is frequently used to sanitize hospital materials and municipal wastewater, including chlorine, chlorine-based substances, H_2_O_2_, ethanol, and quaternary ammonium compounds (QACs) [59]. When vaporized H_2_O_2_ is used for surface disinfection, silver ions can be added. After two weeks of treatment with 30% vaporized H_2_O_2_ mixed with silver ions, hospital rooms treated with this agent were found to be free of methicillin-resistant *Staphylococcus aureus* (MRSA) regrowth [68]. Biswas et al. [69] found that H_2_O_2_ is most effective against resistant *Acinetobacter baumannii* in comparison to NaCl and ClO_2_.

Physical methods are much more difficult to construct. A considerable amount of DNA damage can be caused by UV-C light (wavelength 200–280 nm) that is difficult to repair [70]. According to Rastogi et al. [71] and Schwaz & Schwarz [72], this is a successful disinfection technique used on hospital surfaces. It was reported that the transportable Hyper Light P3 disinfection robot uses UV-C to kill MDR pathogens in hospitals, but that it is only effective when it is within one meter of the patient, with a diminished efficacy at two and three meters [73]. In order to prevent bacteria from growing on medical equipment, silver nanoparticles are frequently used [74]. A sanitization solution comprising zinc oxide nanoparticles could be used to sanitize hospital surfaces polluted with *Pseudomonas aeruginosa*-resistant strains, as suggested by Omrani and Fataei [75]. Chen et al. [76] demonstrated that filters coated with silver nanoparticles and embedded with titanium dioxide eradicated 88% of bacteria in 30 min.

Dunnill et al. [77] also demonstrated that silver nanoparticles and titanium dioxide films could work as a photo-catalyst to kill bacteria under visible indoor light. It is also possible to effectively treat biomedical waste water with nanoparticles and bacteriophages [78]. To treat wastewater effectively, nanoparticle concentrations must be used with caution. Wang et al. [64] have demonstrated the possibility of horizontal transmission of antimicrobial resistance genes between bacteria using zinc oxide nanoparticles at low concentrations. Additionally, metallic nanoparticles have been shown to reduce antimicrobial resistance genes and eliminate microorganisms [79].

### 2.2. Medical Devices and Equipment

Medical devices and equipment carry biological material that can contaminate and spread HAIs [80,81,82]. In several studies, various non-invasive, reusable medical devices, including blood pressure cuffs, splints, breast pumps, basins, pulse oximeter sensors, electrocardiographic (ECG) telemetry systems, and bed handsets, have been identified as potential sources of contaminants [83]. Furthermore, two systematic evaluations demonstrated that reusable, non-invasive medical equipment was very contaminated, with a significant portion also containing pathogenic or drug-resistant organisms [84]. An observational study has also shown evidence of pathogen transmission between medical equipment and patients in healthcare settings [84].

Therefore, keeping medical implants and gadgets sterile is essential to preventing infection in patients. The antimicrobial properties of nanomaterials are used by plants and insects to defend themselves against harmful bacteria [2]. As an example, geckos’ skin is coated with nanoparticles that resemble hair, and they are 200 nm long [85]. Cicadas’ wings are also coated with nano-needles [85]. As soon as these surfaces come into contact with microorganisms, they rupture their cell walls and destroy them [86,87]. These materials tend to be very effective in combating bacteria with Gram-negative walls due to their relatively thin walls. The research community has nevertheless developed a variety of synthetic nanostructured protective coatings that are also fatal to Gram-positive bacteria, drawing inspiration from naturally occurring bactericidal nanoscale materials [2].

### 2.3. Medical Providers’ Personal Belongings and Clothes

Healthcare providers can occasionally make errors when taking off protective clothes when caring for infected patients, resulting in the distribution of multidrug-resistant pathogens to their clothing and equipment. It has been demonstrated that there are resistant bacteria on hospital materials and staff apparel despite strict handwashing practices [88]. Moreover, Michael et al. [89] reported that these microorganisms could also infect laundry rooms, washers, and dryers, resulting in infection (e.g., *Klebsiella pneumoniae* producing extended-spectrum β-lactamases in a 40-bed rehabilitation facility in Holland caused an infection resulting from an infected washer. The infection was controlled after the machine was taken out of service and the guidelines for its use were tightened [90]). Several factors play a critical role in the effective removal of contaminants from textiles and clothes [91], including water temperature, chlorine application, and drying methods. In order to completely eradicate bacteria, washers are recommended to wash at 60 °C [92].

A health care worker’s cellular phone use could expose the community to resistant germs [93]. It appears that this issue is less severe in European countries due to the lower number of resistant germs found on phones [94,95]. The cleaning procedures for smartphones should, however, be followed globally in a stringent manner. It is acceptable to use either in-house or home laundering methods to clean healthcare worker (HCW) uniforms [96]. It is imperative that these procedures are taught to staff, along with precise washing instructions about temperature and chemical additions. Furthermore, HCWs wearing uniforms at work and home raises further concerns. There is no way to dismiss the possibility that bacteria can be acquired from the environment on the way to work as well as the possibility that infectious particles can be distributed from uniforms back to the environment after leaving work. It was found that routinely handled surfaces in East and West London hospitals and community centers carried multidrug-resistant staphylococci [97]. By touching surfaces that are regularly handled, or even by taking public transportation to and from work, one can come into contact with resistant pathogens on clothes [98].

## 3. Currently Available Technologies for Fast Diagnostics of AMR

AMR represents one of the healthcare system’s prevalent problems, making it essential to develop rapid antibiotic sensitivity screening technologies [99]. Clinical microbiology traditionally uses time-consuming, costly, and delivery-intensive approaches and tools to diagnose AMR and perform antimicrobial susceptibility testing (AST). Therefore, presumptive antibiotic therapy is recommended, which has resulted in an increase in death rates and medical expenditures associated with AMR [99]. This has resulted in a growing demand for fast, inexpensive, and economical diagnostic tools for the detection of AMR. These diagnostic tools will drastically shorten the time it takes to determine antibiotic susceptibility, which will allow a choice of improved, target-specific medicines [100].

Infection prevention and control (IPC) programs depend heavily on rapid diagnostic tests for infectious diseases. In addition to reducing mortality, shortening hospital stays, and lowering healthcare costs, rapid diagnostic tests have also demonstrated their effectiveness in improving patient outcomes [101,102]. The current review will highlight some of the most promising non-traditional AST approaches. A number of rapid technologies are being used in the field today, including sequencing, Fourier transform infrared (FTIR), peptide mass fingerprinting technology (e.g., matrix-assisted laser desorption/ionization-time of flight mass spectrometry, MALDI-TOF MS), and lab-on-a-chip.

### 3.1. Sequencing

A number of DNA nucleotide bases were read every day using the primary DNA sequencing techniques, established in the middle of the 1970s. There were two approaches that were most commonly used at that time: the chain terminator [103] and the chemical cleavage process [104]. The results of polyacrylamide gel electrophoresis were able to be resolved to a single base for each response. *Haemophilus influenza* (1,830,137 bp), was the first genome sequenced by an automated sequencer based on fluorescent chemistry using the Sanger method in 1995 [105]. Up until 2005, Sanger sequencing dominated sequencing technologies. It was possible to obtain long, high-quality DNA sequences using these first-generation sequencing techniques despite their limited capacity. The merging of multiple capillaries onto one device allowed for the sequencing of various samples, enabling the sequencing of each sample independently. Multiplexed next-generation sequencing (NGS) was the most important technological advancement of NGS, as it allowed for concurrent evaluation of hundreds of samples. In the NGS technology, DNA is extracted, segmented, ligated to adapters, replicated, and sequenced [99].

The second generation of short-read sequences is flawed due to redundancy, sequence-dependent biases, and repetition faults. On the other hand, pyrophosphate sequencing detects light production and pyrophosphate production, as opposed to Sanger sequencing, which terminates the chain with dideoxynucleotides [99]. Ilumina’s systems, which employ synthetic techniques and integrate fluorescently labeled irreversible terminating sequences into DNA strands for visualization via fluorescent illumination, were recently amended to accomplish the same objective [106]. A third-generation sequencing technique, by contrast, was developed by Pacific Biosciences in 2011, and based on an optical technique combined with a zero-mode waveguide on a nanomaterials technology, it uses a single molecule for long-read sequencing in real time. Another technique was developed by Oxford Nanopore Technologies, which monitors the change in an electrical signal associated with the base that is passing the nanopore as DNA molecules pass through it [99]. As a result of these recently developed second- and third-generation sequencing technologies, complex microbe communities can be described, AMR determinants can be detected, and single genome sequencing can be performed. By sequencing whole metagenomes and genotyping patient samples, we can identify antimicrobial resistance genes from clinical samples without requiring earlier isolation of specific bacteria.

A variety of techniques for sequencing bacterial sequences have been developed, making it easier to access information about bacterial sequences. Enhanced computational models, continuous cost reductions, and fierce industry competition have made sequencing an effective and inexpensive method of antimicrobial resistance gene detection, characterization, and monitoring. Recent studies have used a variety of techniques, instruments, and datasets to discover genetic variations associated with AMR using whole genome sequencing [107] and whole metagenome sequencing data [108]. By combining these technologies with traditional culture-based approaches, we can detect resistance to cultivable and non-cultivable bacteria quickly and accurately. There are two important studies [109] that provide additional details on the use of datasets for AMR diagnosis.

### 3.2. Peptide Mass Fingerprinting Technology

Using MALDI-TOF MS as a peptide mass fingerprinting technology is considered one of the industry’s leading quick microbial detection methods; microorganisms can be identified within minutes by comparing their protein profiles with a library [110,111]. Detecting AMR can also be achieved using MALDITOF MS rather than conventional genotype or phenotypic identification of microorganisms [112,113,114]. In MALDI-TOF MS, spectral fingerprints and profiles can be produced based on intracellular proteins that identify organisms based on genus, species, and subspecies [115,116]. There are a number of MALDI-TOF MS systems available on the market including the MALDI Biotyper from Bruker Daltonics Bremen and VITEK MS from bioMérieux, Marcy l’Étoile. A comparison of the performance of the two platforms has been conducted in the literature [117,118,119]. There are more than 300 types of bacteria and yeasts in the Bruker MALDI Biotyper, which was approved by the FDA in 2018 [120,121]; this tool has been validated by the FDA.

In order to find antibiotic mechanisms of resistance, such as carbapenems, MALDI-TOF MS is now being used [122], but standardization is important to ensure reliable results [123]. There are many advantages to MALDI-TOF MS [124,125,126], including reliability, speed (within minutes), consistency, accessibility, economy, and environmental sustainability. In spite of the fact that MALDI-TOF MS has significantly reduced the time it takes to detect microorganisms and advances have been made in the identification of AMR [127], these devices are prohibitively expensive (purchase and maintenance) and are large enough to serve as an indication of AST systems in limited laboratories in healthcare settings.

### 3.3. Spectroscopy-Based Approaches

Methods such as surface-enhanced Raman scattering (SERS) are considered the principal biochemical fingerprinting methods, because they accurately reflect bacterial molecular profiles and changes brought about by antimicrobial treatment [128,129,130]. Several studies have used SERS to investigate bacteria’s tolerance to antibiotics or the spread of antibiotic resistance, along with analyzing how drugs work by looking at the entire cell’s spectroscopic signature [131,132]. By using SERS, it can detect pathogenic strains rapidly, with extreme sensitivity, and with a minimal amount of sample processing [128,133]. Based on the study previously described by Lu et al. [134], SERS can also be applied to microfluidic chips that combine SERS and methicillin sensitive *Staphylococcus aureus* for quick detection and MRSA differentiation.

SERS still has some drawbacks, despite recent improvements in its specificity and sensitivity in bacterial biosensors [135]. It is typically necessary to dry samples before analysis, which may lead to consistency problems. When identifying pathogens in the liquid phase, there is usually difficulty interrogating cells in their natural habitat as a consequence of scattering from the Raman laser source. An additional constraint for SERS is the sample and the experimental settings, i.e., laboratory testing is typically conducted on samples containing only one bacterial species. Further advances in the determination of molecular spectral patterns (such as nucleobases) have not yet been made in the development of datasets representing the SERS spectra of macromolecules nor in the statistical analysis and interpretation of spectra [136]. It should eventually be possible to identify multiple pathogens from a complex sample using microbial SERS biosensors.

## 4. Innovative Therapeutic Approaches

The treatment and control of mild to severe diseases has been the goal of antimicrobial drugs for many years. In the late 1920s, penicillin was accidentally introduced, and many improvements were made to the revolutionary drug [137]. Although standard antimicrobial therapies have failed in the past two decades to control the various types of multidrug-resistant bacteria in the healthcare settings, no new category of antibiotics has been developed [138]. As a result, scientists were motivated to discover new ways to combat these problems. Recently, certain diseases (e.g., AIDS) that were previously incurable have also been treated with new antiviral medications developed by research studies. The use of antiparasitic and antifungal medications has also become a crucial component of IPC in healthcare sectors.

Currently, 13 antibiotics are undergoing Phase II clinical trials and 13 are undergoing Phase III clinical trials [139]. There are several novel classes of antibiotics, but most of them are adaptations or assemblages of current antibiotic classes. Gepotidacin is the first medication in a unique synthetic class of triazaacenaphthylene (C_9_H_5_N_3_) bacterial topoisomerase inhibitors, used for urogenital gonorrhea and simple urinary tract infections. It has now moved into Phase III clinical trials after eradicating *Neisseria gonorrhoeae* in 95% of patients in Phase II clinical trials. The Swiss company Polyphor AG also manufactures murepavadin (a new synthetic class antibacterial). *Pseudomonas aeruginosa* is treated effectively with this drug, especially in people with cystic fibrosis since it blocks lipopolysaccharide uptake by bacteria. After conducting a Phase III clinical study for an intravenous dose in 2018, the medication was stopped in 2019 because of a higher prevalence of acute renal failure among participants [140]. Additionally, Polyphor obtained approval for a Phase I trial to test murepavadin’s effectiveness against *Pseudomonas aeruginosa* in cystic fibrosis patients in December 2020, with a Phase II trial starting soon (Polyphor, 2021).

In order for an antibiotic to reach the market it usually takes ten to fifteen years and about US $1.5 billion after it is initially discovered [141]. Moreover, large pharmaceutical corporations are wary of entering the market with an antibiotic due to its limited and constrained use after approval, and the likelihood that resistance can develop during its use. The result has been the departure of a number of important stakeholders from the pharmaceutical market [142]. In the current review, this section examines and evaluates the methods that are being used or recommended as alternatives to conventional antibiotics.

### 4.1. Nano-Sized Particles Approach to Multidrug-Resistant Bacteria

The term nanoparticles (NPs) refers to particles having a diameter between 1 and 100 nanometers [143]. NPs are increasingly being used as bacterial growth inhibitors, for instance, as coatings for implants and medical materials. Additionally, they are capable of serving as effective antimicrobial agents [138,144]. However, other metals show antibacterial properties only in the NP form, whereas other bulk metals have antibacterial effects against both Gram-positive and Gram-negative bacteria [145]. Three strategies have been postulated to happen in order, albeit the precise mode of action via which NPs exhibit their antimicrobial activities is currently not thoroughly grasped. Oxidative stress, metal ion production, and non-oxidative processes are among these. It is primarily through these mechanisms that (1) bacteria’s outer membranes break down, and/or their cell walls are destroyed, (2) intracellular and extracellular elements are contacted with NP-derived ions, (3) the use of photocatalytic activity to create reactive oxygen molecules that harm microbial structures, (4) DNA synthesis is suppressed, (5) enzymatic activity is suppressed, and (6) energy transmission is disrupted [138].

According to Lee et al. [146] metal nanoparticles are responsible for interfering with a number of metabolic pathways, including membrane rupture, cytochrome suppression, ribosome instability, and DNA fragmentation. It has been shown that metal NPs have the ability to prevent the growth of *Salmonella typhi*, *Staphylococcus aureus* (including methicillin-resistant strains), *Escherichia coli*, *Pseudomonas aeruginosa*, *Salmonella enterica*, and *Klebsiella pneumoniae*. Furthermore, antibiotic-coated nanomaterials have shown synergistic activity against drug-resistant microorganisms (e.g., gold NPs coated in vancomycin enhanced their ability to kill Vancomycin-resistant Enterococcus). The study of Muzammil et al. [147] indicated that a combination of zinc nanoparticles and beta-lactams has proven effective against various types of microorganisms such as ESBL-producing *Klebsiella pneumoniae*, *Pseudomonas aeruginosa*, and *Escherichia coli* isolates, which normally cause urinary tract infections. Due to their pharmacological properties, NPs can be used to treat bacteria by inhaling, orally administering, applying topically, and injecting intravenously. There is a serious concern about the toxicity of nanoparticles when employed as new antimicrobials [148,149]. Researchers have evaluated both the in vitro and vivo effects of nanoparticles on human and animal organs and tissues. When NPs are present in quantities that hinder bacterial growth, they are dangerous to living cells. The distribution of NPs to specific infection sites may reduce toxicity but may affect the NP’s potential [150,151].

### 4.2. The Monoclonal Antibody Approach to Multidrug-Resistant Bacteria

In recent years, monoclonal antibodies (mAbs) have gained popularity as an effective treatment for everything from tumors to infectious diseases [152]. It is widely recognized that monoclonal antibodies can treat a wide range of disorders [153] and they make a great therapeutic choice in this area. The FDA approved muromonab-CD3 as the first monoclonal antibody for use in therapy [154]. Permission was granted in 1985 for it to be used in treating organ rejections after transplantation. In July 2021, 100 mAbs had been approved by the FDA [155]. A growing interest in monoclonal antibodies is often attributed to their well-tolerated character, specialized nature, and lack of off-target effects [156]. Producing and developing mAbs is viable when drug companies use strong platform procedures [157]. A number of promising mAbs have already been identified as being effective at controlling disease as a result of significant improvements in bioinformatic techniques and genome and proteomics research, enabling a molecular understanding of diseases and microorganisms [152]. Among the five types of antibodies (IgA, IgM, IgD, and IgE), IgG is the most commonly used for therapeutic purposes. Molecular engineering can be carried out with it due to its long half-life, high abundance in serum, and application in protein engineering [152].

In light of the emerging antibiotic resistance issue in many pathogenic microorganisms, monoclonal antibodies have attracted increasing interest as an alternative anti-bacterial treatment method because of their role in promoting human defense against bacteria [158]. Unrestricted antimicrobial use and globalization are causing a dramatic increase in AMR, which could make some bacteria untreatable in the near future. The concept of antibody-based therapy may be gradually developed as a means of combating AMR in diseases that are difficult to treat [159].

### 4.3. Bacteriophages (Phages)

It is possible to replace antimicrobial therapy with bacteriophages (phages) in order to fight diseases caused by bacteria that are resistant to several different types of antibiotics [160]. The latest generations of studies have involved both human and animal models, using phages and antibiotics individually or in combination [161]. Drugs may cause host bacteria to create phages when they are cooperated with phages. There are many benefits to phage therapy compared to antibiotics. The phage reproduces inside host bacterial cells, assuming the characteristics of bacterial infections, being extremely specialized for one species or even one strain of pathogen [162]. As an interesting fact, phages are prevalent throughout the surrounding environment, from the water system to the oil and sewage systems. Despite the fact that AMR mechanisms do not hinder their effectiveness, they do not require additional dosages as do antibiotics, because they occur naturally at the infection site [163]. In recent studies, it has been suggested that bacteriophages can be used as an alternative to antibiotics because of their self-replication ability, high immunity with fewer adverse effects on eukaryotic cells, and their ability to maintain their activity in numerous environmental settings [164,165].

In addition to their antimicrobial efficacy, phages are allegedly superior to antimicrobials. As phages can only multiply inside bacterial cells and cannot enter mammalian cells, they are considered to be significantly safer and more effective than conventional antibiotics. According to Kakasis and Panitsa [166], all perspectives coming from eastern Europe and most recent experiments on humans and animals have not revealed any substantial adverse consequences following bacteriophages administration. It is also easier to administer phages because their effects last longer in the human body, e.g., for many days, unlike antibiotics, which require repeated injections quickly after one another over a period of several days [167]. In many cases, only a few doses are needed, since the phage concentration rises after the first delivery. When compared with antibiotics, they only work on those parts of the infection that can be reached, even if the bacteria are found in organs or systems where antimicrobials have difficulty getting to [168]. There is no doubt that phages can help combat the rise of bacterial resistance to antibiotics, and some study findings suggested that this would be a wise course of action. It is limited adequate clinical trials assessing phage efficacy today, and the results of these trials are inconsistent. The development of formulations for clinical use in bacterial control, the prevention or reduced likelihood of antibiotic-resistant bacteria emergence, as well as the prevention of genetic material transmission are all open questions [168]. Bacteria and phages coevolve, but the mechanism governing this evolution is still unknown.

### 4.4. Antimicrobial Peptides (AMPs)

As an alternative to antibiotics, the application of AMPs could prove to be one of the most exciting treatments. This can help combat an array of bacterial infections, especially those caused by multidrug-resistant pathogens [169]. A large number of AMPs have been identified, each with a unique structure and mode of action, giving them a unique biological activity [169,170]. It has been widely recognized more than two decades ago that AMPs are extremely promising since they are present in nature for long periods of time without showing signs of resistance [171]. They are particularly attractive because they do not experience rapid resistance development as do antibiotics [169]. Due to AMPs’ variety of mechanisms of action against bacteria as opposed to antibiotics’ fixed targets, resistance against microorganisms is unlikely to develop rapidly or easily [172]. A significant advantage of AMPs over other treatments is that they are broken down into amino acids as opposed to producing hazardous byproducts as do other treatments [169]. It is generally accepted that all living organisms possess AMPs, which are tiny polypeptide molecules that contain between 12 and 50 amino acids on average [173]. As secondary metabolites, these compounds play an important role in the body’s innate defense system. In vertebrates, these compounds are usually synthesized by epithelial cells; however, they can also be produced by phagocytic cells.

A large number of commensal and pathogenic microorganisms are associated with these peptides, which can be found in both tissues and mucous membranes. As a result of these peptides, several pathogens, including viruses, protozoans, bacteria, and fungal species, may be suppressed or destroyed [174,175]. A number of convergent anti-biofilm effects of AMPs has been demonstrated in previous studies. These effects include interfering with or deteriorating the membrane permeability of cells embedded within biofilms, disrupting bacteria’s signaling mechanisms, degrading polysaccharide and biofilm matrix, inhibiting the alarming system for preventing the microbial strict reaction, and reducing gene expression involved in biofilms and protein transport [176]. The use of AMPs, therefore, has been utilized as a means of preventing the growth of biofilms and removing those that are already established. Since standard antibiotics have a very low metabolic activity, they are not capable of eradicating persistent bacterial forms. Accordingly, AMPs have been extensively researched as one of the most promising approaches to eliminating these persistent bacterial forms, demonstrating excellent inactivation capabilities [177].

### 4.5. The Vaccine’s Potential Role in Fighting Multidrug-Resistant Pathogens

A vaccination strategy protects both humans and animals from illness and outbreaks by using fewer medicines and by limiting the spread of resistant microorganisms [138]. The role of vaccines in fighting AMR is twofold: directly by reducing the risk of infection, and indirectly by stopping the spread of AMR-resistant strains to other species. Infection rates and secondary infections that would otherwise require excessive antibiotic treatment are decreased when antibiotic prescription is decreased [178,179]. The decrease in resistance spread is one of the most effective weapons against AMR when herd immunity and vaccination strategies are implemented [180]. As reverse vaccinations became more widespread, creating effective vaccines against MDR microorganisms became relatively easy [181,182]. Researchers are working on developing vaccines against resistant strains of *Escherichia coli*, *Pseudomonas aeruginosa*, and *Klebsiella pneumoniae* and *Staphylococcus aureus* [21,179].

While significant progress is being made in this field, vaccines against the main antibiotic-resistant diseases remain a long way off. New possibilities are opening up for the development of vaccines against microorganisms, including antibiotic-resistant ones, with major advances in bioinformatics and omics sciences. It is hypothesized that vaccination against asymptomatic carriers of microorganisms that repopulate the nasal cavity, mucous membrane, and digestive system, as well as other places, such as *Streptococcus pneumoniae*, *Staphylococcus aureus*, and Enterobacteriacae, minimizes the diversity of bacteria, thereby reducing resistance genetic transfer [183]. In spite of the fact that vaccines don’t replace antibiotics, they are able to aid us in reducing antimicrobial resistance by lowering the amount of antibiotics we use, preventing illnesses caused by AMR bacteria, and halting the spread of AMR bacteria to others.

## 5. Conclusions

In order to combat antibiotic resistance, different approaches are needed. Identifying infectious agents accurately and rapidly will improve medical professionals’ ability to treat certain infections on site. Modern infection control technologies will also make it possible to manage multidrug-resistant infections efficiently. Vaccination will decrease antibiotic usage and stop new resistance mechanisms from emerging. It is also important to improve the hygiene habits of patients and healthcare professionals to reduce the likelihood of multidrug-resistant bacteria escaping into the environment in the future. These measures will improve hospital decontamination as well as sewage treatment. It will also be necessary to overcome future restrictions due to the fact that some infections have no breakpoints for particular antibiotics. Academics and pharmaceutical companies cannot alter the direction of events on their own, despite all the developments. There is a need for government intervention to inform the population and persuade big pharmaceutical companies to work on antibiotic development again. A closer look at the use of antibiotics is necessary in all nations, and public education campaigns such as the WHO’s World Antibiotic Awareness Week are needed to reach a wider audience. Using our resources efficiently and working together can make a huge difference in defeating multidrug-resistant pathogens in healthcare settings.

## Data Availability

Not applicable.
